# Time–frequency time–space LSTM for robust classification of physiological signals

**DOI:** 10.1038/s41598-021-86432-7

**Published:** 2021-03-25

**Authors:** Tuan D. Pham

**Affiliations:** Center for Artificial Intelligence, Prince Mohammad Bin Fahd University, Khobar, 31952 Saudi Arabia

**Keywords:** Physiology, Diseases, Mathematics and computing

## Abstract

Automated analysis of physiological time series is utilized for many clinical applications in medicine and life sciences. Long short-term memory (LSTM) is a deep recurrent neural network architecture used for classification of time-series data. Here time–frequency and time–space properties of time series are introduced as a robust tool for LSTM processing of long sequential data in physiology. Based on classification results obtained from two databases of sensor-induced physiological signals, the proposed approach has the potential for (1) achieving very high classification accuracy, (2) saving tremendous time for data learning, and (3) being cost-effective and user-comfortable for clinical trials by reducing multiple wearable sensors for data recording.

## Introduction

Analysis and classification of clinical time-series data in physiology and disease processes are considered as a catalyst for biomedical research and education. Innovative computerized tools for physiological data classification are increasingly needed to facilitate investigations on new unsolved challenging problems in clinical and life sciences with respect to both basic and translational perspectives. Conventional methods for classification of physiological time series to detect abnormal conditions include fractals, chaos, nonlinear dynamics, signal coding, pattern matching, and machine learning. The current surge of modern artificial intelligence (AI) opens a new approach for sequential data classification with long short-term memory (LSTM) networks^1^, which are an architecture of deep learning. LSTM networks are a type of recurrent neural networks that learn order dependence in sequential data.

There are many methods developed for classification of time series in different fields of applications. Time-series classification algorithms based on discriminatory features can be categorized into six main groups^2^: (1) whole series, (2) intervals, (3) shapelets, (4) dictionary, (5) combinations, and (6) model. For the whole-series approach, classification is performed by comparing the similarity between two time series using a distance measure. The methods of intervals choose one or multiple intervals of the series and use summary measures as features for classification. The methods of shapelets define a class with phase-independent patterns called shapelets, then a class is identified by the existence of one or more shapelets in the whole time series. The dictionary-based methods classify time series based on the frequency of its recurring subseries. The methods of combinations try to combine two or more methods of the whole series, intervals, shapelets, and dictionary for classification. The model-based methods fit a time series to mathematical models constructed for the classes and then assign the time series to the class that has the largest similarity score given by the class model. Most recently, deep-learning methods or deep neural networks have been reported to outperform many baseline time-series classification approaches and appear to be the most promising techniques for classifying temporal data^3^.

Because LSTM networks can capture long-term temporal dependencies, they have been applied to provide solutions for many difficult problems in bioinformatics and computational biology^4^. As a state-of-the-art method for learning physiological models for disease prediction, many applications of LSTM and other deep-learning networks have recently been reported in literature, such as classifying electroencephalogram (EEG) signals in emotion, motor imagery, mental workload, seizure, sleep stage, and event related potentials^5^, non-EEG signals in Parkinson’s disease (PD)^6^, learning and synthesis of respiration, electromyograms, and electrocardiograms (ECG) signals^7^, decoding of gait phases using EEG^8^, and early prediction of stress, health, and mood using wearable sensor data^9^.

The present work presents a time–frequency time–space LSTM tool for robust and efficient classification of physiological time series, while solutions obtained from conventional LSTM networks would result in lower accuracy and higher data training time. Furthermore, for the case of clinical gait analysis with the use of measurement sensors to assess biomechanical patterns and therapeutic plan for rehabilitation in patients disabled from conditions such as PD and post stroke, long walk trials are recommended to obtain at least 370 strides^10^. Such long-distance walks result in long records of physiological measurements, cause discomfort to the patients, and may be impractical to perform in many clinical settings^11^.

Differentiating patients with PD from healthy controls using gait data was studied in^12^, which trained fuzzy neural networks with wavelet features extracted from the gait data. Another study extracted gait features with the short-time Fourier transform and used the support vector machines (SVMs) for the classification task^13^. To capture the local changes in the dynamics of gait signals, the feature-extraction method of shifted 1-D local binary patterns and a multilayer perceptron, which is a class of feed-forward artificial neural networks, were used for the classification of PD and healthy controls^14^. The extraction of time-domain and frequency-domain features of gait data for training with random decision forests, which are an ensemble machine-learning method for classification, was reported in a more recent study for detecting patients with PD^15^. All these studies employed shallow neural networks or SVMs. However, deep neural networks are known to be the most advanced models of the neural-network approach and shown to be of performance superior to other types of statistical classifiers^16^.

The novel idea for classification of physiological data with LSTM presented herein is the creation of complementary time–frequency and time–space features of time series. In signal processing, instead of viewing a time series as a one-dimensional signal, time–frequency analysis studies a signal in both time and frequency domains simultaneously by some function whose domain is the two-dimensional real plane to extract transient features from the signal by a time–frequency transform. Time–frequency signal processing for feature extraction was reviewed as a useful approach for pattern recognition^17^ that provided successful applications, including EEG seizure detection and classification^17^, classification of ultra-high-frequency signals^18^, classification of vibration events^19^, and classification of EEG signals and episodic memory^20^.

In nonlinear dynamics, the time–space analysis attempts to transform one-dimensional signal into a two-dimensional space to enable the visualization of the recurrences of states of a dynamical system at certain times and enable the extraction of distinctive features representing behaviors of different dynamical mechanisms underlying nonlinear time series. The extraction of novel features from time series not only facilitate the power of signal compression for deep learning but also enhances the capability of LSTM networks for robust signal classification. In chaos theory, the method of recurrence plots (RPs) was developed for nonlinear time-series analysis^21^. RPs and extended methods were further addressed for the analysis of complex systems^22,23^ and dynamical features of nonlinear time series^24^. While an RP is a binary visualization of recurrences of states of a dynamical system at certain pairs of time, a fuzzy recurrence plot (FRP)^25^ displays the visualization as a grayscale image. Because of being much richer in texture than RPs, the technique of FRPs of time series is a preferred approach for texture analysis and has been successfully applied to extract texture features for pattern recognition, including classification of PD and control subjects using deep learning^6,26^, tensor decomposition^27^, and SVMs^28^; and other neuro-degenerative diseases^29^.

In general, the time–frequency analysis is known a preferred approach for the representation and essential feature extraction of non-stationary signals because it is effective for estimating the underlying characteristics composing the signals^17^, whereas the time–space analysis provides another kind of visual information about the signals by detecting hidden dynamical features being inherent in the data. The combination of complementary features generated by both time–frequency and time–space analysis methods is therefore promising for enhancing the classification power of the sequential deep learning.

## Data

### ECG data

ECG signals capture the electrical activity of a human heart over a period of time. ECG signals are used by physicians for examining the condition of a patient’s heartbeat to detect if the condition is normal or irregular. Atrial fibrillation (AF) is a type of irregular heartbeat that occurs when the upper chambers of the heart (atria) beat out of coordination with the lower chambers (ventricles). The ECG data^30^ used in this study are publicly available from the *PhysioNet: The Research Resource for Complex Physiologic Signals*. The data consist of ECG signals sampled at 300 Hz and classified by a group of experts into normal sinus rhythm, AF, alternative rhythm, and noise. The purpose of the creation of this challenging database was to call for the development of new methods for classifying these types of cardiac arrhythmias. Information about the number of participants in the recordings of normal rhythm, AF rhythm, and other rhythms is not available from the data source^31^.

### Gait in Parkinson’s disease data

The Gait in Parkinson’s Disease database^32^ consists of time series of vertical ground reaction force in Newtons of gait dynamics from 93 patients with idiopathic PD and 73 healthy controls. This database is also publicly available from the *PhysioNet: The Research Resource for Complex Physiologic Signals*. The data consist of the vertical ground reaction force (in Newtons) signals of the subjects as they walked at their usual, self-selected pace for approximately 2 minutes on level ground. The force was measured as a function of time with 8 sensors placed underneath each foot. The force signals of each of the 16 sensors placed under the two feet of each subject were digitized and recorded at 100 samples per second.

## Time–frequency and time–space analysis

### Instantaneous frequency

The instantaneous frequency (IF) of a non-stationary signal is a time-varying parameter that relates to the average of the frequencies *f* present in the signal as it evolves over time instants *t*^33,34^. The IF function estimates the IF of a signal at a sampling rate by computing the spectrogram power spectrum *P*(*t*, *f*) and estimating the IF as

1 $$\begin{aligned} IF(t) = \frac{\int _{-\infty }^{\infty } f P(t,f) df}{\int _{-\infty }^{\infty } P(t,f) df}. \end{aligned}$$

The power spectrum is a mathematical expression of the amount of the signal at a frequency *f*. For a periodic signal, peaks at the fundamental frequency and its harmonics are observed at the spectrum; for a quasiperiodic signal, peaks at linear combinations of related frequencies observed; and a chaotic signal yields broad band components to the spectrum. In practice, the exact solution for the power spectrum cannot be determined because a signal *x*(*t*) is not infinitely long but measured over a finite interval $0 \le t \le T$. Therefore, the power spectrum needs to be numerically estimated. A method for estimating the power spectrum of a time series $x_k$, $k = 0, \dots , N-1$ is described as follows.

The spectral density of a time series of length *N* can be approximated as^35^

2 $$\begin{aligned} P_N(f) = \frac{\Delta t}{N} \left|\sum _{k=0}^{N-1} x_k e^{-i 2 \pi fk \Delta t} \right|^2, \end{aligned}$$

where $\Delta t$ is the sampling interval.

If the spectral value is calculated at $f = j \Delta f$, where $\Delta f = 1/(N \Delta t)$, and $\Delta t = 1$, then

3 $$\begin{aligned} P_j = \frac{1}{N} \left|\sum _{k=0}^{N-1} x_k e^{-i 2 \pi \frac{jk}{N}} \right|^2 = \frac{1}{N} \left|X_j \right|^2, \end{aligned}$$

which indicates the discrete Fourier transform (DFT), $X_j$, as

4 $$\begin{aligned} X_j = \sum _{k=0}^{N-1} x_k e^{-i 2 \pi \frac{jk}{N}}, j = 0, \dots , N-1. \end{aligned}$$

However, it was proved that the power spectrum estimate expressed in Eq. (3) is not properly scaled^35^. Therefore, the estimate is modified as

5 $$\begin{aligned} P_j= & {} \frac{1}{WN} \left|\sum _{k=0}^{N-1} w_k x_k e^{-i 2 \pi \frac{jk}{N}} \right|^2, j = 0, \dots , N-1; \end{aligned}$$

in which

6 $$\begin{aligned} W = \frac{1}{N} \sum _{j=0}^{N-1} w_j^2, \end{aligned}$$

where $w_j$, $j= 0, \dots , N-1$, are the weights or coefficients of a window function (the Kaiser window^36^ is applied in this study).

The estimate of $P_j$ expressed in Eq. (5) using the fast Fourier transform (FFT) can be sequentially carried out as follows^35^.

- Truncate the time series or pad with zeros so that $N=2^n$, where *n* is a positive integer.
- Weight the time series with a window function.
- Calculate the DFT of the weighted time series $(w_k x_k)$ using the FFT.
- Calculate $P_j$ using Eq. (5).

### Spectral entropy

The spectral entropy (SE) of a signal is a measure of its spectral power distribution^33,34^. The SE treats the normalized power distribution of the signal in the frequency domain as a probability distribution and calculates its Shannon entropy. The Shannon entropy in this context is known as the spectral entropy of the signal. Given a time–frequency power spectrogram *P*(*t*, *f*), the probability distribution at time *t*, $0 \le t \le T$; and frequency point *m*, $m = 1, \dots , N$; denoted as *p*(*t*, *m*), is

7 $$\begin{aligned} p(t,m) = \frac{P(t,m)}{\sum _f P(t,f)}, \end{aligned}$$

where $f \in [0, fs/2]$ is specified in this study, and *fs* is the sampling frequency.

The spectral entropy at time *t*, denoted as *H*(*t*), is given as

8 $$\begin{aligned} H(t) = - \sum _{m=1}^N p(t,m) \log _2 p(t,m). \end{aligned}$$

### Fuzzy recurrence plot

In the study of dynamical systems, a sequence of values in time can be transformed into an object in space. This transformation allows the sequence to be analyzed in space. Such space is called the phase space. The object in the phase space is called the phase space set. The transformation of a sequence of values in time into an object in the phase space can be done using the time-delay embedding^37^. The embedding dimension describes the space (such as a line, an area, or a volume) that contains the object^38^. Time delay, which is also called lag, expresses the amount of offset in a time series. Mathematically, the phase-space reconstruction using time-delay embedding for a time series ($z_1, z_2, \dots , z_I$) can be performed as ${{\mathbf {y}}}_i = (z_i, z_{i+\phi }, \dots , z_{i+(d-1)\phi }$, $i = 1, \dots , I-(d-1)\phi$, where $\phi$ and *d* are time delay and embedding dimension, respectively.

In fuzzy logic^39^, a fuzzy set is defined as a collection of distinct objects whose membership grades in the set are expressed with real numbers. In mathematic terms, let *U* be a universe of discourse and *F* a subset of *U*. The fuzzy set *F* is characterized by a fuzzy membership function $\mu _F(x)$ that maps each element $x \in U$ to the interval [0, 1]: $\mu _F(x): U \rightarrow [0, 1]$. The real value of $\mu _F(x)$ is called the fuzzy membership grade of *x* in *F*. The notion of a fuzzy set can be expressed in the following three cases: 1) $\mu _F(x) = 0$ if *x* is not totally in *F*, 2) $\mu _F(x) = 1$ if *x* is totally in *F*, and 3) $0< \mu _F(x) < 1$ if *x* is partially in *F*. Thus, the greater value of the fuzzy membership grade is, the more certain *x* is a member of *F*.

In cluster analysis, data points can be assigned to different groups or clusters. Points that are most similar to each other belong to the same cluster. Based on the concept of fuzzy sets, fuzzy clustering assigns the data points to all clusters with different degrees of fuzzy membership. In other words, the fuzzy membership value of a data point for a certain cluster indicates how positive the data point belongs to that cluster.

Now let ${{\mathbf {X}}} = ({\mathbf {x}}_1, \dots , {\mathbf {x}}_N) \in {\mathbb {R}}^{Nm}$ with ${\mathbf {x}}_i \in {\mathbb {R}}^m$ be a phase-space collection of a signal transformed by the time-delay embedding method, *c* a pre-defined number of clusters, ${{\mathbf {V}}}=\{{\mathbf {v}}_1, \dots , {\mathbf {v}}_c\}$ a set of clusters, and $\mu ({\mathbf {x}}_i,{\mathbf {v}}_q)$, $i=1, \dots , N$, $q=1, \dots , c$, fuzzy membership grades expressing the degrees of phase-space vectors ${\mathbf {x}}_i$ belonging to cluster centers ${\mathbf {v}}_q \in {{\mathbf {V}}}$. These fuzzy membership grades can be determined using the fuzzy *c*-means algorithm^40^. An FRP, denoted by $\tilde{{\mathbf {R}}}$, is defined as^25^

9 $$\begin{aligned} \tilde{{\mathbf {R}}}(i,j) = \mu ({\mathbf {x}}_i,{\mathbf {x}}_j), \, i, j = 1, \dots , N, \end{aligned}$$

where $\mu ({\mathbf {x}}_i,{\mathbf {x}}_j) \in [0, 1]$ is the fuzzy membership of similarity between ${\mathbf {x}}_i$ and ${\mathbf {x}}_j$.

The elements of an FRP, $\tilde{{\mathbf {R}}}(i,j)$, $i = 1, \dots , N$, $j = 1, \dots , N$, can be inferred using three properties of fuzzy relations as follows.

1. Reflexivity:
  10 $$\begin{aligned} \mu ({\mathbf {x}}_i,{\mathbf {x}}_i) = 1, \, i=1, \dots , N. \end{aligned}$$
2. Symmetry:
  11 $$\begin{aligned} \mu ({\mathbf {x}}_i,{\mathbf {v}}_q) = \mu ({\mathbf {v}}_q,{\mathbf {x}}_i), \, i = 1, \dots , N, q = 1, \dots , c. \end{aligned}$$
3. Transitivity:
  12 $$\begin{aligned} \mu ({\mathbf {x}}_i,{\mathbf {x}}_j) = \max [\min \{\mu ({\mathbf {x}}_i,{\mathbf {v}}_q), \mu ({\mathbf {x}}_j,{\mathbf {v}}_q)\}], q = 1, \dots , c. \end{aligned}$$

**Figure 1:**
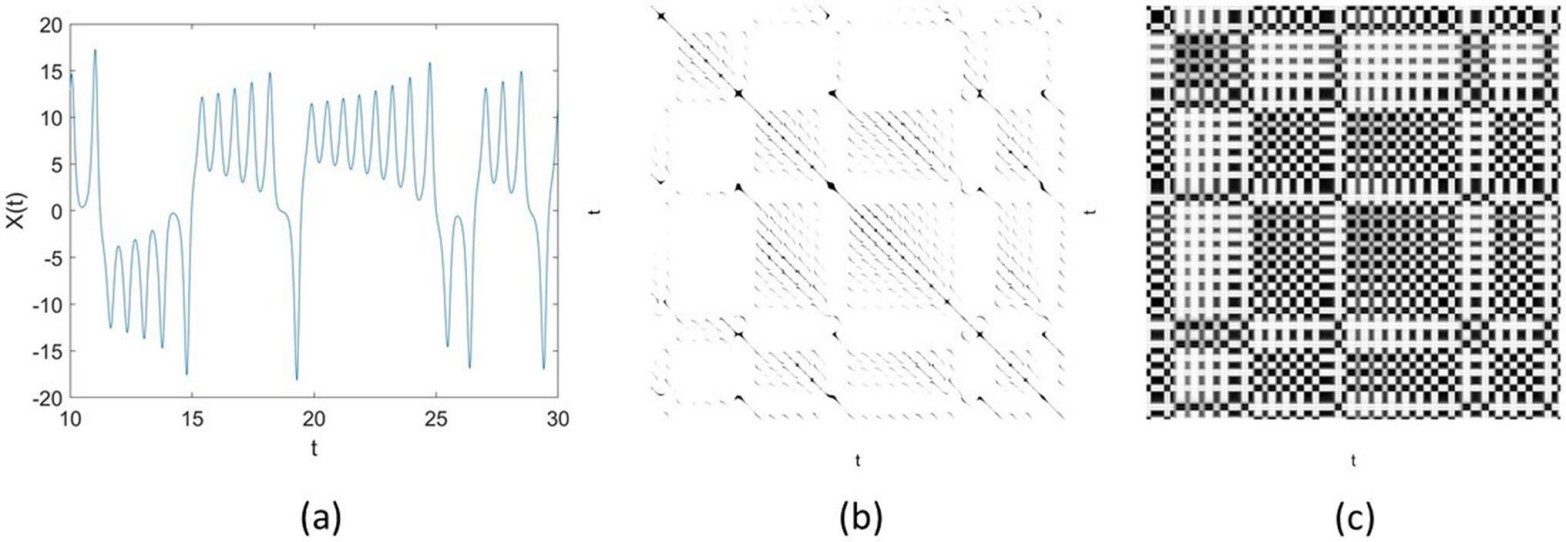
Recurrence of a dynamical system: (**a**) time series of the Lorenz *X*-component, (**b**) recurrence plot, and (**c**) fuzzy recurrence plot.

As an example, to illustrate some difference in the visual display of an RP and an FRP, Fig. 1 shows a time series of 2000 points of the *X*-component of the Lorenz (chaotic) system^41^, and its RP and FRP. The RP was constructed using the embedding $= 3$, time delay $= 1$, and a conventional value for the similarity threshold $= 5\%$ of the standard deviation of the signals. The FRP was constructed using the embedding $= 3$, time delay $= 1$, and number of clusters $= 3$. The grayscale image of the FRP is much richer in texture than the binary image of the RP.

### Fuzzy recurrence image entropy

Entropy of a grayscale image is a statistical measure of randomness to characterize the texture of the image. As an FRP is a grayscale image, the entropy of an FRP image is defined as

13 $$\begin{aligned} E_{FRI} = - \sum _{k=1}^{K} p_k \log _2 p_k, \end{aligned}$$

where $K = 256$, which is the number of gray levels of the FRP (obtained by converting real values of pixels in [0, 1] to integers in [0, 255]), and $p_k$ is the probability associated with the intensity level *k*, $k = 1, \dots , K$, obtained from the normalized histogram for the *k*-th bin.

### Fuzzy recurrence entropy

Based on the definition of the non-probabilistic entropy of a fuzzy set^42^, the entropy of an $N \times N$ FRP or fuzzy recurrence entropy that is a measure of the degree of uncertainty of recurrences of the reconstructed phase space of a signal is defined as^43^

14 $$\begin{aligned} E_{FR} = \sum _{i=}^N \sum _{j=1}^N - \mu ({\mathbf {x}}_i,{\mathbf {x}}_j) \, \log _2 \mu ({\mathbf {x}}_i,{\mathbf {x}}_j) - [1-\mu ({\mathbf {x}}_i,{\mathbf {x}}_j)] \, \log _2[1-\mu ({\mathbf {x}}_i,{\mathbf {x}}_j)], \end{aligned}$$

where $\mu ({\mathbf {x}}_i,{\mathbf {x}}_j)$ corresponds to $\tilde{{\mathbf {R}}}(i,j)$ defined in Eq. (9).

## Time–frequency time–space long short-term memory networks

**Figure 2:**
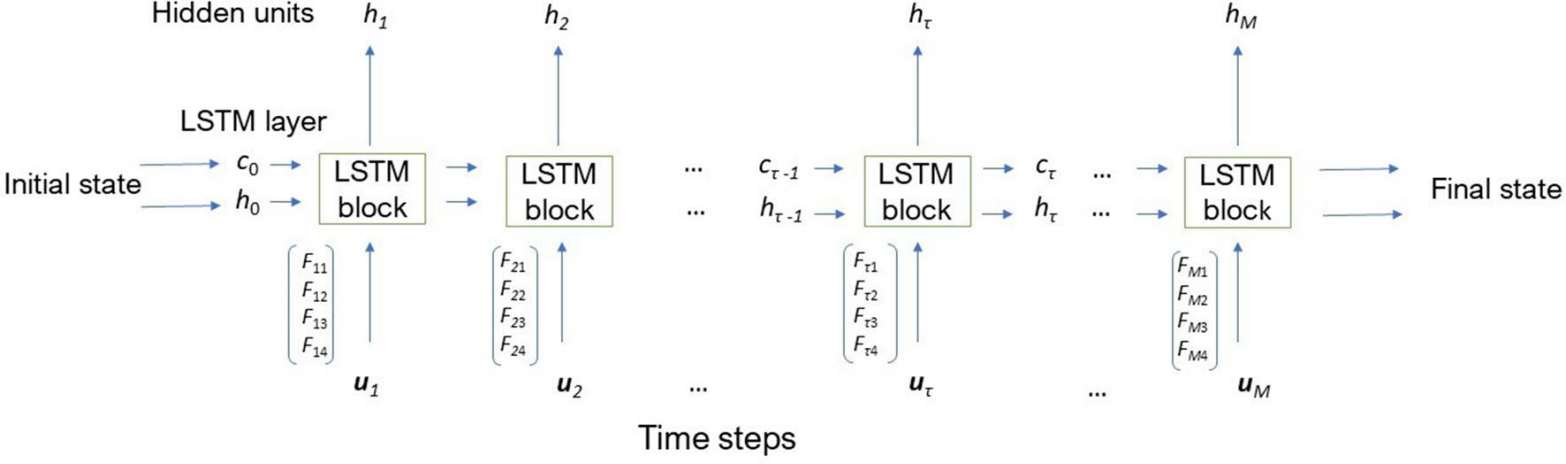
TF–TS LSTM layer architecture.

Based on LSTM networks^1,4,44^, in which the proposed input time–frequency (TF) and time–space (TS) features are included, the architecture for a TF–TS LSTM block is graphically described in Fig. 2. This figure illustrates the flow of an input time series ${{\mathbf {u}}} = ({{\mathbf {u}}_1}, \dots , {{\mathbf {u}}_M}) \in {\mathbb {R}}^{MQ}$ through an LSTM layer, where *M* is the number of segments split from the original time series of length *L*, and *Q* the number of features. In this study, $M = \lceil L/N \rceil$, where $N=128$, $\lceil \rceil$ denotes the ceiling function, and $Q=4$. The input at a time point is the concatenation of the four features extracted for the segment at the same time point, i.e., ${{\mathbf {u}}}_\tau = (F_{\tau 1}, F_{\tau 2}, F_{\tau 3}, F_{\tau 4})^T$, $\tau = 1, \dots , M$, where $F_{\tau 1}$, $F_{\tau 2}$, $F_{\tau 3}$, and $F_{\tau 4}$ are the instantaneous frequency, spectral entropy, fuzzy recurrence image entropy, and fuzzy recurrence entropy extracted from segment ${{\mathbf {u}}}_\tau$, respectively.

The learnable weights of an LSTM layer are the input weights, denoted as ${{\mathbf {a}}}$, recurrent weights, denoted as ${{\mathbf {r}}}$, and bias, denoted as *b*. The matrices ${{\mathbf {A}}}$, ${{\mathbf {R}}}$, and vector ${{\mathbf {b}}}$ are the concatenations of the input weights, recurrent weights, and bias of each component, respectively. The concatenations are expressed as

15 $$\begin{aligned} {{\mathbf {A}}} \, = \, [{{\mathbf {a}}}_{i}, {{\mathbf {a}}}_{f}, {{\mathbf {a}}}_{g}, {{\mathbf {a}}}_{o}]^T, \end{aligned}$$

16 $$\begin{aligned} {{\mathbf {R}}} \,= \, [{{\mathbf {r}}}_{i}, {{\mathbf {r}}}_{f}, {{\mathbf {r}}}_{g}, {{\mathbf {r}}}_{o}]^T, \end{aligned}$$

17 $$\begin{aligned} {{\mathbf {b}}} \,= \, [{b}_{i}, {b}_{f}, {b}_{g}, {b}_{o}]^T, \end{aligned}$$

where *i*, *f*, *g*, and *o* denote the input gate, forget gate, cell candidate, and output gate, respectively.

The cell state at time step $\tau$ is defined as

18 $$\begin{aligned} {{\mathbf {c}}}_\tau = f_\tau \circ {{\mathbf {c}}}_{\tau -1} + i_\tau \circ g_\tau , \end{aligned}$$

where $\circ$ is the Hadamard product.

The hidden state at time step $\tau$ is given by

19 $$\begin{aligned} {{\mathbf {h}}}_\tau = o_\tau \circ \sigma _c({{\mathbf {c}}}_{\tau }), \end{aligned}$$

where $\sigma _c$ is the state activation function that is usually computed as the hyperbolic tangent function (tanh).

At time step $\tau$, the input gate ($i_\tau$), forget gate ($f_\tau$), cell candidate ($g_\tau$), and output gate ($o_\tau$) are defined as

20 $$\begin{aligned} i_\tau \,=\, \sigma _g ({{\mathbf {a}}}_i {{\mathbf {u}}}_\tau + {{\mathbf {r}}}_i {{\mathbf {h}}}_{\tau -1} + {b}_i), \end{aligned}$$

21 $$\begin{aligned} f_\tau \,=\, \sigma _g ({{\mathbf {a}}}_f {{\mathbf {u}}}_\tau + {{\mathbf {r}}}_f {{\mathbf {h}}}_{\tau -1} + {b}_f), \end{aligned}$$

22 $$\begin{aligned} g_\tau \,=\, \sigma _c ({{\mathbf {a}}}_g {{\mathbf {u}}}_\tau + {{\mathbf {r}}}_g {{\mathbf {h}}}_{\tau -1} + {b}_g), \end{aligned}$$

23 $$\begin{aligned} o_\tau\,=\, \sigma _g ({{\mathbf {a}}}_o {{\mathbf {u}}}_\tau + {{\mathbf {r}}}_o {{\mathbf {h}}}_{\tau -1} + {b}_o), \end{aligned}$$

where $\sigma _g$ denotes the gate activation function that usually adopts the sigmoid function.

A bidirectional LSTM (bi-LSTM)^45^ is an extension of traditional LSTM that can improve performance on sequence classification problems. Instead of being trained with one LSTM on the input time series, a bi-LSTM architecture is trained with both time directions simultaneously with hidden forward and backward layers. The first on the input time series as it is and the second on a reversed copy of the time series. This architecture learns bidirectional long-term dependencies between time steps of time series and therefore can provide additional context to the network and result in fuller learning on the data.

**Figure 3:**
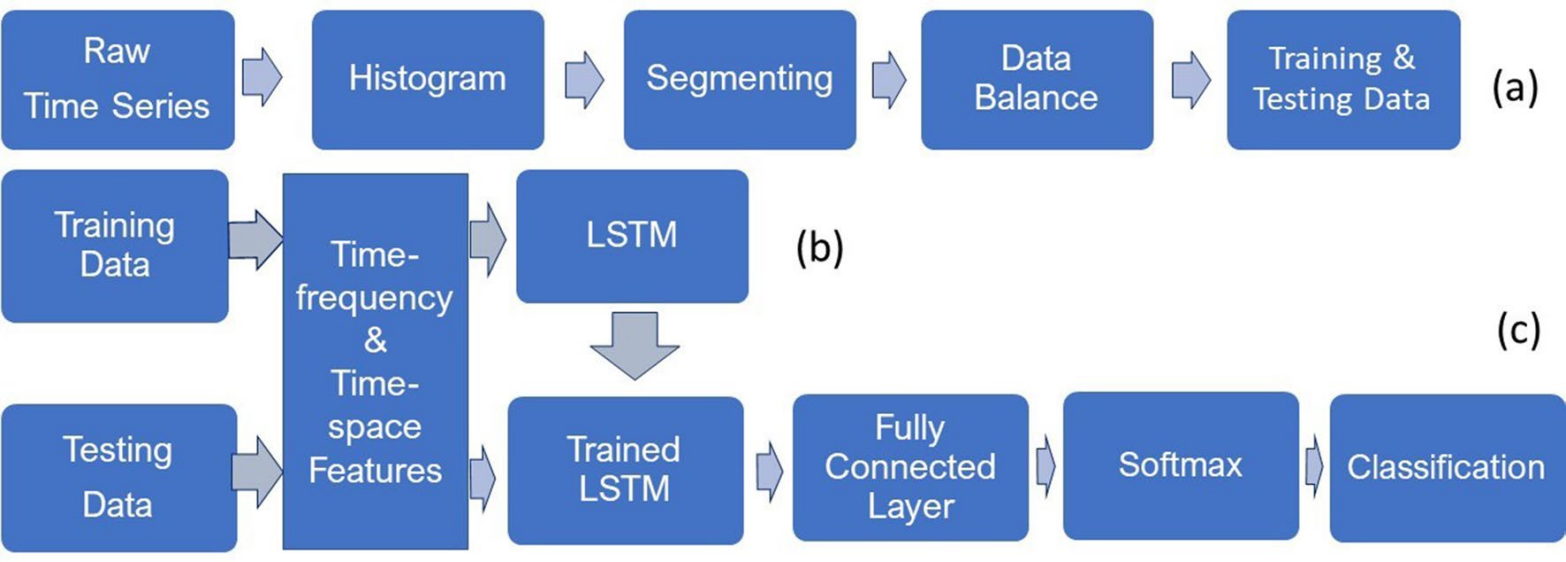
Procedure for classification of physiological time series with TF–TS LSTM: (**a**) from raw data to data balance in training and testing, (**b**) transformation of raw time series into time–frequency and time–space features, and (**c**) classification of testing data.

The procedures for obtaining data balance for training and testing sets, and the transformation of raw time series into TF and TS features for LSTM learning and classification are outlined in Fig. 3.

To obtain signals of the same length contained in both training and testing datasets, the histogram of the distribution of the lengths of the signals is observed to detect the majority length. Signals of lengths that are less than the majority are discarded, and those that are longer than the majority are split into segments of the majority length and the remaining samples of the signal are ignored if there are any. Creating signals of equal length is particularly useful for the training of the networks that breaks the data into mini-batches. In the same mini-batch, the training pads or truncates the signals to have the same length. However, it is known that the process of padding or truncating can reduce the performance of the networks because of the added or missed information caused by the padding or truncating, respectively. To obtain the data balance in each class for both training and testing, copies of the signals of the minority class are repeated to achieve the same size of the signals of the majority class. This step is described in Fig. 3a. The next step is to extract the TF features of the signals using the instantaneous frequency and spectral entropy and the TS features of the signals using the fuzzy recurrence image entropy and fuzzy recurrence entropy for training the networks (Fig. 3b). The same TF and TS features are extracted from the testing signals as the input for the trained TF–TS LSTM networks to carry out the classification task (Fig. 3c).

## Performance measures

Let condition positive *P* be the total number of disease signals, condition negative *N* the total number of healthy control signals, true positive *TP* the number of disease signals correctly identified as disease, false positive *FP* the number of healthy control signals incorrectly identified as disease, true negative *TN* the number of healthy control signals correctly identified as healthy control, and false negative *FN* the number of the disease signals incorrectly identified as healthy control.

Accuracy (*ACC*) is defined as

24 $$\begin{aligned} ACC = \frac{TP+TN}{P+N}. \end{aligned}$$

Sensitivity (*SEN*) is defined in this study as the portion of the disease signals that are correctly identified as having the condition:

25 $$\begin{aligned} SEN = \frac{TP}{P}. \end{aligned}$$

Specificity (*SPE*) is the portion of the healthy control signals that are correctly identified as not having the disease:

26 $$\begin{aligned} SPE = \frac{TN}{N}. \end{aligned}$$

Precision (*PRE*) is calculated as

27 $$\begin{aligned} PRE = \frac{TP}{TP+FP}. \end{aligned}$$

$F_1$ score is the harmonic mean of precision and sensitivity and calculated as

28 $$\begin{aligned} F_1 = \frac{2TP}{2TP+FP+FN}. \end{aligned}$$

## Results

Tables 1 and 2 list the tenfold cross-validation results of two physiological databases: ECG, and Gait in Parkinson’s Disease, respectively. For the ECG database, this experiment used normal sinus rhythm (5050 signals) and AF (738 signals) for binary classification. For the Gait in Parkinson’s Disease data, this study used the time series recorded from only one sensor under the left foot labeled as L5 on the database. The purpose of selecting the sensor data recorded at the L5 location was to compare with the work reported in^47^, which used four sensors at L5, L7, R7, and R8 for the classification of gait patterns. The LSTM used in this study was the bi-LSTM (LSTM will be used as bi-LSTM subsequently). To extract the TF features, sampling frequency was set as 300 Hz. To extract the TS features, the embedding dimension $= 1$, time delay $= 1$, and number of clusters $= 3$ for computing the FRPs. The specifications of the FRP parameters were based on previous studies^25,43^, which provided satisfactorily results and were not as sensitive for constructing FRPs as for RPs^25^.

**Table 1:** Ten-fold cross-validation metrics for ECG of classification of atrial fibrillation and normal sinus rhythm.

Method	Sensors	% ACC	% SEN	% SPE	% PRE	$F_1$	Time (mins)
**ECG of atrial fibrillation and normal sinus rhythm**							
LSTM	1	$58.37 \pm 0.01$	$82.86 \pm 0.03$	$33.88 \pm 0.09$	$55.62 \pm 0.06$	$0.67 \pm 0.02$	$3506 \pm 19$
TF–TS LSTM	1	$\mathbf{93.77} \pm 0.07$	$91.43 \pm 0.05$	$96.12 \pm 0.01$	$95.93 \pm 0.01$	$0.94 \pm 0.02$	$\mathbf{1} \pm 0.11$

LSTM $=$ direct use of time series by bi-LSTM, TF–TS LSTM $=$ use of time–frequency and time–space features of time series by bi-LSTM.

**Table 2:** Ten-fold cross-validation metrics for classification of gait of patients with Parkinson’s disease and healthy controls.

Method	Sensors	% ACC	% SEN	% SPE	% PRE	$F_1$	Time (mins)
Lee and Lim^12^	8	77.33	81.10	65.48	n/m	n/m	n/m
Daliri^13^	16	91.20	91.71	89.92	n/m	n/m	n/m
Ertugrul et al.^14^	16	88.89	93.93	77.66	n/m	n/m	n/m
Acici et al.^15^	16	98.04	99.10	95.70	n/m	n/m	n/m
Zeng et al.^47^	4	96.99	96.77	97.26	n/m	n/m	n/m
LSTM	1	$78.95 \pm 0.03$	$100.00 \pm 0.00$	$60.00 \pm 0.10$	$69.23 \pm 0.05$	$0.82 \pm 0.04$	$111 \pm 9.95$
TF–TS LSTM	1	$100.00 \pm 0.00$	$100.00 \pm 0.00$	$100.00 \pm 0.00$	$100.00 \pm 0.00$	$1 \pm 0.00$	$< 1$

LSTM $=$ direct use of time series by bi-LSTM, TF–TS LSTM $=$ use of time–frequency and time–space features of time series by bi-LSTM, and n/m $=$ not mentioned.

All TF and TS features were standardized to improve the network training and testing^46^. For the LSTM specifications, the network layer with an output size $= 100$, fully connected layer $= 2$ (two classes), followed by a softmax layer and a classification layer. Training options of the bi-LSTM were set as optimizer $=$ ‘Adam’ (adaptive moment estimation), including $L_2$ regularization factor, maximum number of epochs $= 80$, minimum batch size $= 150$, initial learning rate $= 0.01$, and gradient threshold $= 1$.

For the ECG data, the TF–TS LSTM significantly outperformed conventional LSTM in terms of classification accuracy (58% and 94% for conventional LSTM and TF–TS LSTM, respectively), other statistical measures (sensitivity, specificity, precision, and $F_1$ score), and training time (3506 minutes and 1 minute for LSTM and TF–TS LSTM, respectively, where the time for computing the four features was excluded in the TF–TS LSTM training). The specificity (34%) is much lower than the sensitivity (83%) obtained from the conventional LSTM, while these two measures are much more balanced using the TF–TS LSTM (sensitivity $= 91\%$ and specificity $= 96\%$).

For the gait data, using the signals recorded from only one sensor, TF–TS LSTM provided perfect classification metrics (accuracy $= 100\%$, sensitivity $= 100\%$, specificity $= 100\%$, precision $= 100\%$, and $F_1$ score $= 1$) with the training time of $< 1$ minute (the time for computing the four features was excluded). The use of conventional LSTM yielded the accuracy $= 79\%$ with 111 minutes for data training. Other five previous methods^12–15,47^ that studied the same database used the number of sensors between 4 and 16 obtained accuracy rates between 77%^12^ and 98%^15^ (standard deviations of classification results obtained from these five methods were not given in literature^47^).

## Discussion

**Figure 4:**
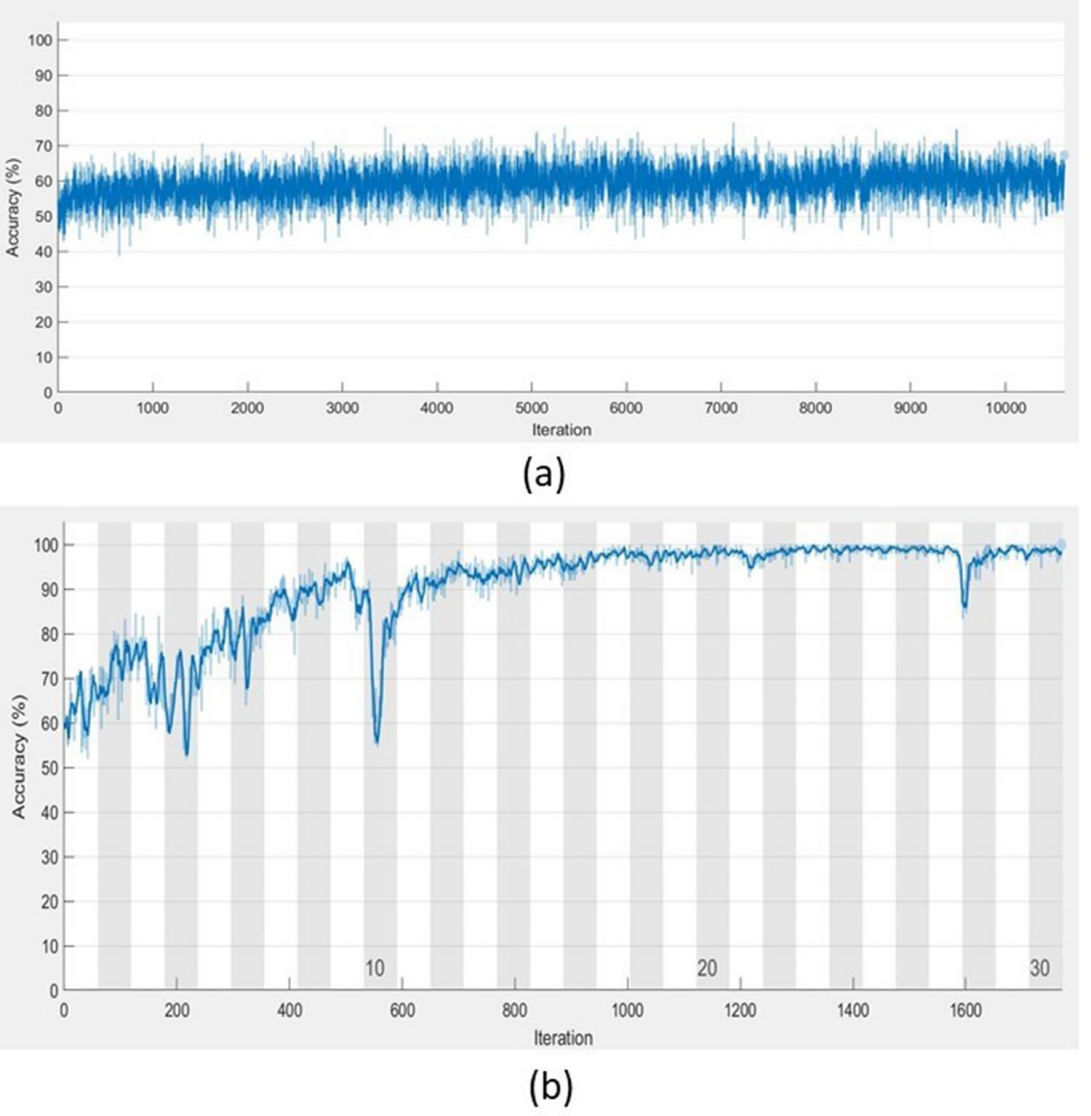
Training processes of (**a**) bi-LSTM and (**b**) TF–TS bi-LSTM, using tenfold cross-validation of the ECG data.

Computer experiments have shown that TF–FS LSTM achieved very high performance in the classification task and saved tremendous training time in comparison with the conventional implementation of the conventional LSTM. As an example, Fig. 4 shows the contrast of the training processes of conventional LSTM and TF–TS LSTM with respect to the convergence of accuracy and the number of iterations. Not only the TF–TS LSTM outperformed conventional LSTM, classification results of gait in Parkinson’s disease in terms of accuracy, sensitivity, specificity, precision, and $F_1$ score obtained from the TF–TS LSTM are higher than those previously reported in literature. In particular, the TF–TS LSTM used the data recorded from only one sensor. The significant reduction in biomedical sensors to measure human physiological parameters in real time for disease detection has an implication for promising the user’s comfort and contributing to the low cost, simplicity, and portability in wearable sensor technology.

In this study, only the gait data recorded by one sensor located at L5 were used to compare with the other work^47^ that included the data recorded by four sensors located at L5, L7, R7, and R8. The gait classification with the use of a single sensor located at L5 obtained from the proposed TF–TS LSTM outperformed the use of the four sensors for the classification obtained from the methods of phase space reconstruction, empirical mode decomposition, and neural networks^47^. Tests of the TF–TS LSTM for the gait classification using data recorded from other single sensors were not carried out. However, the current comparison has shown the better performance of the TF–TS LSTM. As the five methods^12–15,47^, which were compared with the TF–TS LSTM using the gait data, were proposed and implemented by other authors, it would be difficult to fairly implement these methods for the classification of the ECG data without the provision of the source codes. However, it is shown that the test results obtained from the TF–TS LSTM are significantly higher than the LSTM using the two datasets, and the classification accuracy obtained from the LSTM using the gait data from only one sensor (79%) is higher than the result reported in^12^ using the gait data from 8 sensors (77%).

Here the signal lengths were made to be the same length of the majority. In case, if the majority does not exist or the histogram has a uniform distribution, the signal lengths can be made to be equal to the length of the shortest signal. In general, signals of lengths that are shorter than the majority can be included for the classification. However, it has been mentioned earlier, creating signals of equal length can be more effective for the network training and testing. In practice, the recording of physiological signals that meet some standard length for testing is feasible because it is based on the majority.

As shown in Fig. 4, the high accuracy of the TF–TS LSTM training could be reached while the training of the LSTM with raw time series could not improve much in accuracy. Furthermore, the TF–TS LSTM requires much shorter time for training in comparison with the training of raw long time series. This is because it is trained with sequential features of the time series instead of the time series, where the length of the features is much shorter than that of the original data and the effectiveness of the standardized features is an important factor for improving the network performance during training.

Feature extraction can be related to dimensionality reduction by which multivariate data can be reduced to lower-dimensional space for more manageable data processing. The physiological time series used in this study are one-dimensional time series. On the contrary, these time series were split into equal segments from which the four features were extracted for learning and classification by the TF–TS LSTM. In other words, the one-dimensional time series were transformed into much shorter sequences of 4 feature dimensions as shown in Fig. 2. The extracted features provide essential information of the data in time–frequency and time–space domains, which are intended to be complementary, informative, and non-redundant responses. Thus, the transformed data can facilitate the subsequent learning and leverage discriminative power of the sequential deep learning, leading to better class predictions. The results obtained in this study have shown the TF–TS LSTM outperformed other statistical classifiers, including SVMs and multilayer perceptron.

In summary, the finding is that training the LSTM network with raw time series produce poor classification results but training the network with TF and TS features extracted from the signals can both significantly enhance the classification performance and reduce the training time.

The Matlab-based TF–TS LSTM software for classification of physiological signals is designed to be easily utilized by biomedical and life science users who do not have technical knowledge in AI, signal processing, and general physics by following provided step-by-step instructions (Supplementary Note). In biomedical data, the problem of data imbalance is common, which can significantly prevent classifiers from achieving good results. The software suggests how to design a balance of class samples for training and testing datasets when minority classes exist.

## Conclusions

An AI-based approach for improving the performance in detecting diseases using physiological signals have been presented and discussed. The proposed method takes advantages of information extracted from both frequency and space out of the temporal data for effective deep learning to increase the classification task and lower computational complexity. Although the method was developed for classifying time series in physiology, it can be readily applied to the classification of other biological and clinical signals, such as time series in gene expression^48^, neurology^49^, and epidemiology^50^.

The AI-based method presented in this work was tested using the records obtained from a single-sensor measurement of gait in PD. The results suggest the method has potential to be able to reduce the need of using multiple sensors for recording physiological data, thus resulting in both cost-saving and comfort to the participants. Further tests of the method with other multiple-sensor data would be necessary to confirm the finding. Wearable sensors are useful devices for evaluating patient outcomes in clinical trials. However, the devices need to provide physical ease to participants so that they are prepared to wear them. Otherwise, the deployment of such tools will not be practically feasible, particularly when applying to the older adult ($> 50 \,\hbox {years}$) population^51^.

## Software availability

MATLAB software, ECG data for AF and normal sinus rhythm, and Supplementary Note for running the ECG data used in this paper are publicly available at the author’s personal homepage: https://sites.google.com/view/tuan-d-pham/codes under the title “TF–TS LSTM”.

## Supplementary Information

Supplementary Information.
