# Supplementary material for: Time–frequency time–space LSTM for robust classification of physiological signals
Source: Sci Rep. 2021 Mar 25;11:6936. doi: 10.1038/s41598-021-86432-7 (PMC7994826; doi:10.1038/s41598-021-86432-7)
Supplement: Supplementary file 1 — Supplementary Information. [file 41598_2021_86432_MOESM1_ESM.pdf]

Time-Frequency Time-Space LSTM for Robust Classification of Physiological Signals

Tuan D. Pham[1,*]

[1]Center for Artificial Intelligence,

Prince Mohammad Bin Fahd University,

Khobar 31952, Saudi Arabia

***Corresponding author (email: tpham@pmu.edu.sa)

Supplementary Note

- This note provides main pieces of information in addition to the in-line comments provided in the main Matlab code "`classify_gait_L5_LSTM.m`"

- Other included codes called by the main code are: "`segmentSignals2.m`", "`frpentropy2.m`"

- Other included codes called by "`frpentropy2.m`" are: "`frp.m`", "`frpFentropy.m`"

- The "`gaitData_L5.mat`" is provided as an example for executing the software. The data contain signals recorded by sensor L5 placed beneath the left foot of patients with PD and control subjects.

- MATLAB R2020a or newer version and its Deep Learning, Signal Processing, Image Processing, and Fuzzy Logic toolboxes are needed to execute the software.

1) Reading data

The given "`gaitData_L5.mat`" file contains two variable names "`Signals`" (165-by-1 cells of PD and control signals) and "`Labels`" (165-by-1 labels for 'Control' and 'PD').

`load gaitData_L5`: read the file named "`gaitData_L5`" to load "`Signals`" and "`Labels`" data.

2) Generate a histogram of signal lengths and observe signals of majority length.

```
L = cellfun(@length,Signals);
h = histogram(L);
```

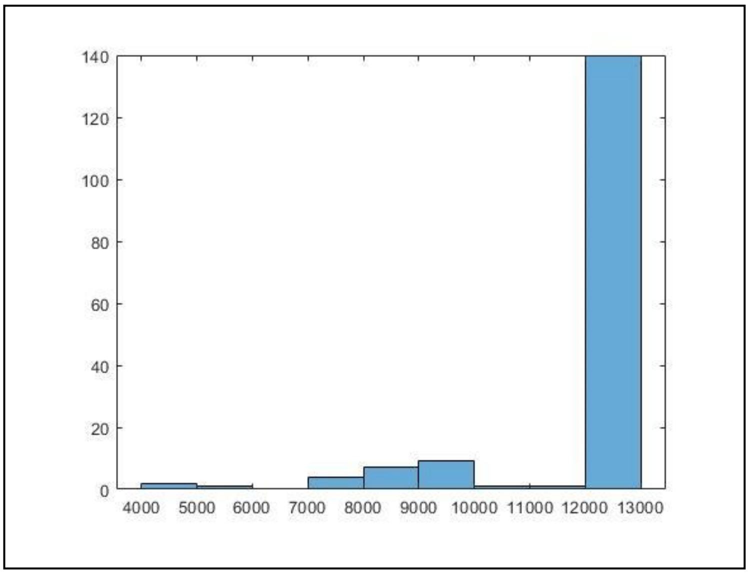

The histogram shows that most signals are of length 12,000 and above. To remove minority signal lengths, signals from 12,000 and above are kept and the others disregarded using the function "`segmentSignals2.m`"

```
N = 12000;
[Signals,Labels] = segmentSignals2(Signals,Labels,N);
```

3) Construct training and testing data for 10-fold cross-validation

```
[trainIndA,~,testIndA] = dividerand(numPD,0.9,0.0,0.1);

[trainIndN,~,testIndN] = dividerand(numControl,0.9,0.0,0.1);
```

Other *k*-fold cross-validations can be specified. For example, for 2-fold cross-validation, change 0.9 to 0.5 and 0.1 to 0.5.

4) Balancing training and testing data

There are 84 PD signals and 42 Normal signals. Use 84 PD signals, and then use the `repmat` function to repeat the first 42 Normal signals 2 times.

```
XTrain = [XTrainA; repmat(XTrainN(1:42),2,1)];
YTrain = [YTrainA; repmat(YTrainN(1:42),2,1)];

XTest = [XTestA; repmat(XTestN(1:5),2,1)];
YTest = [YTestA; repmat(YTestN(1:5),2,1)];
```

Use the summary function to display the number of signals of `Control` and `PD` in training and testing data:

```
summary(YTrain)
summary(YTest)
```

Training:

```
Control   84

PD        84
```

Testing:

```
Control   10

PD        9
```

5) LSTM network architecture and training options are explained by in-line comments of the main Matlab code "`classify_gait_L5_LSTM.m`"

6) Executing the main code "`classify_gait_L5_LSTM.m`" will display the following results and figures:

```
LSTMAccuracy = 100


confMat =

    10     0

     0     9


ACC = 100

SEN = 100

SPE = 100

PRE = 100

F1 =  1

AUC = 1
```

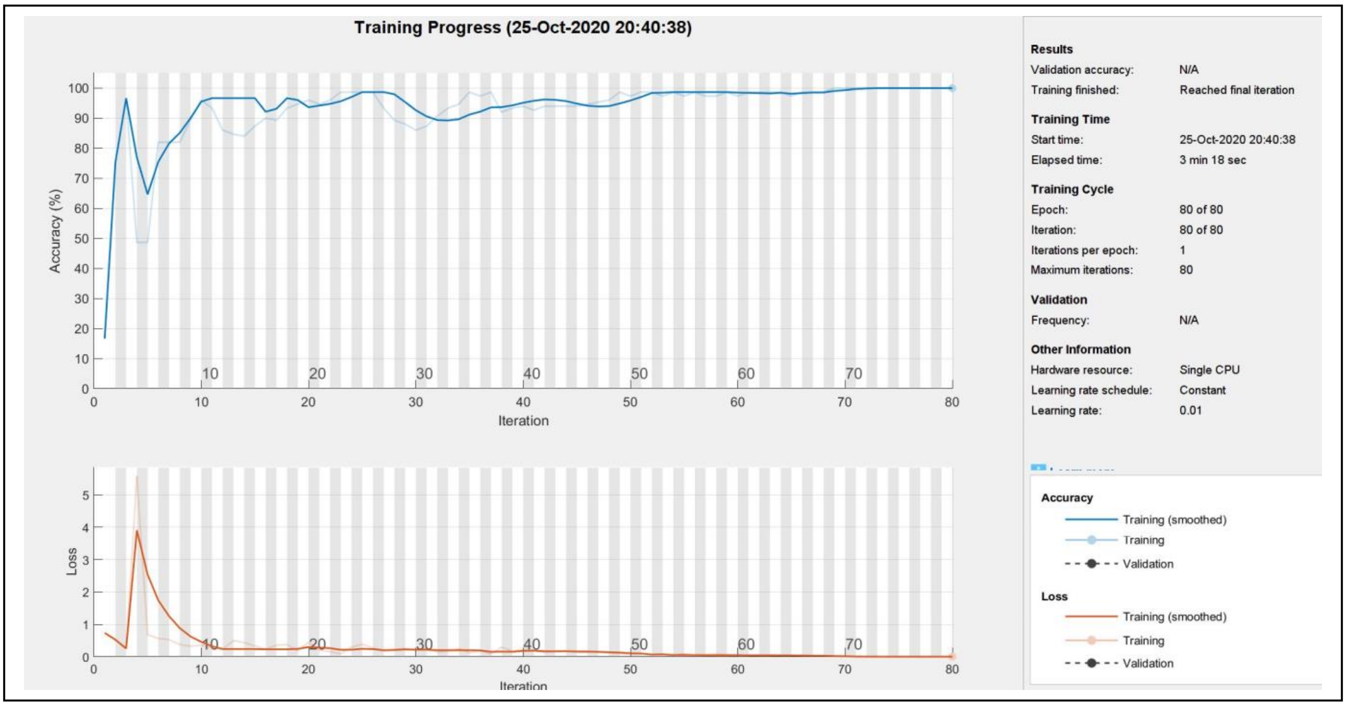
Training Progress (25-Oct-2020 20:40:38)
Accuracy (%)
Iteration
Loss
Results
Validation accuracy: N/A
Training finished: Reached final iteration
Training Time
Start time: 25-Oct-2020 20:40:38
Elapsed time: 3 min 18 sec
Training Cycle
Epoch: 80 of 80
Iteration: 80 of 80
Iterations per epoch: 1
Maximum iterations: 80
Validation
Frequency: N/A
Other Information
Hardware resource: Single CPU
Learning rate schedule: Constant
Learning rate: 0.01
Accuracy
Training (smoothed)
Training
Validation
Loss
Training (smoothed)
Training
Validation

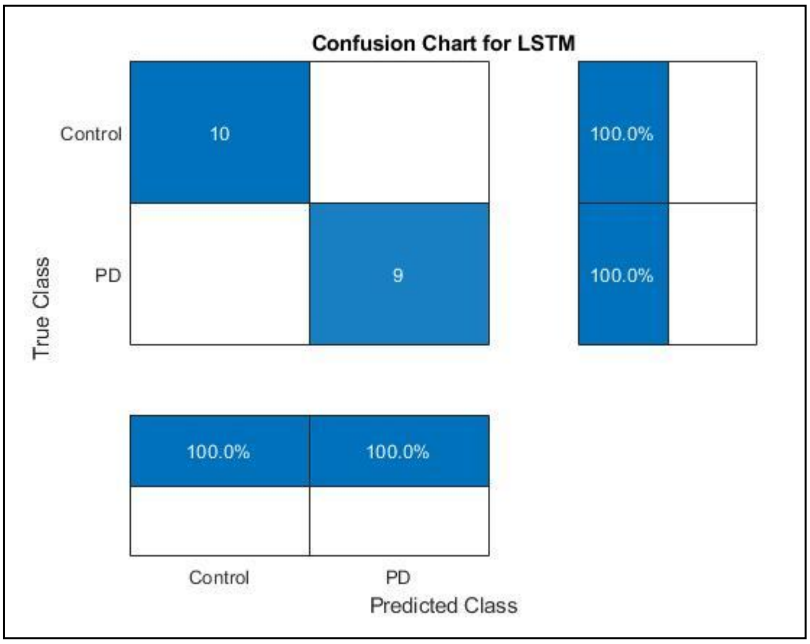
Confusion Chart for LSTM
Control
PD
True Class
10
9
100.0%
100.0%
100.0%
100.0%
Control
PD
Predicted Class
